# Immunomodulatory Effects of Flavonoids in Colitis-Associated Colorectal Cancer

**DOI:** 10.3390/ijms27041883

**Published:** 2026-02-15

**Authors:** Sonia H. Navia, Libia Vega, Tonathiu Rodríguez, Miriam Rodríguez-Sosa

**Affiliations:** 1 Laboratory of Innate Immunity, Research Unit in Biomedicine (UBIMED), Facultad de Estudios Superiores Iztacala, Universidad Nacional Autónoma de México, Av. De los Bariios 1, Los Reyes Iztacala, Tlalnepantla 54090, Mexico; so.navia@ciencias.unam.mx (S.H.N.); tonathiurh@unam.mx (T.R.); 2 Department of Toxicology, Centro de Investigación y de Estudios Avanzados del Instituto Politécnico Nacional, Av. IPN 2508, Zacatenco, Ciudad de México 07360, Mexico; lvega@cinvestav.mx

**Keywords:** ulcerative colitis, colon cancer, flavonoids, inflammation, immuno-oncology

## Abstract

Flavonoids present in plants and fruits have been used in traditional medicine to reduce inflammation under various inflammatory conditions, including colitis. The pharmacological mechanisms that regulate intestinal inflammation are associated with the colonic microbiota, protection against oxidative stress, preservation of epithelial barrier function, and immune homeostasis. This review describes the main flavonoids present in the diet and examines their role in mitigating colon inflammation, as well as their impact when chronic inflammation progresses to colitis-associated colorectal cancer (CAC), in which flavonoids may promote immune evasion and tumor growth. Understanding the effects of flavonoids on colon physiology offers an opportunity to use these compounds rationally in the treatment of colitis and prevention of the development of colorectal cancer (CRC).

## 1. Introduction

Colitis is a chronic inflammatory condition of the colon characterized by a dysregulated immune response against the intestinal microbiota, leading to persistent mucosal inflammation, epithelial damage, and compromised barrier function. In addition to the clinical burden of symptoms such as abdominal pain, diarrhea, and rectal bleeding, which severely compromise patient quality of life, the real danger lies in its long-term progression. A sustained inflammatory environment substantially elevates the risk of developing colorectal cancer over time [1,2]. Flavonoid consumption has been linked to health benefits, including protective effects against diseases associated with intestinal inflammation, alleviation of colitis symptoms, and reduction in CAC progression through anti-inflammatory actions [3]. However, suppressing inflammation could interfere with antitumor immunity, depending on the CRC stage and phenotype. Therefore, the use of flavonoids in the treatment of CAC must be carefully considered.

This review analyzes the role of flavonoids in the regulation of intestinal inflammation and explores how flavonoids regulate intestinal inflammation, modulate immune responses, and influence CAC. In addition, the duality of the effects of flavonoids is discussed, highlighting the need for a balanced approach that considers both their benefits and their possible risks at different stages of the disease. This analysis provides a comprehensive overview that can guide future research and therapeutic strategies for the management of flavonoids as treatments.

This study evaluated the complex interplay between dietary flavonoids and CRC, focusing on their anti-inflammatory properties and their implications across various stages of CRC development. We synthesized established data from databases, including Google Scholar, PubMed, and Scopus, covering the period from January 2000 to June 2025. Our goal is to bridge existing knowledge gaps by examining the cellular and molecular facets of inflammatory processes within the flavonoid-CRC relationship. The search was guided by specific descriptors such as “inflammation,” “flavonoids,” “immune evasion” and “immunological escape” in the context of CRC progression. Our selection process prioritized studies detailing anti-inflammatory mechanisms of action that also function as key immune pathways during different phases of tumor growth. To maintain this thematic focus, we applied strict exclusion criteria, discarding any literature involving research unrelated to the colorectal region or studies on flavonoid anticancer properties not directly linked to inflammatory processes. Following this rigorous screening process, 164 out of the 233 articles consulted were selected for inclusion in this review.

## 2. Chemical Structure of Flavonoids

Flavonoids present in a wide range of sources, such as fruits, vegetables, herbs, spices, legumes, nuts, mushrooms and even plant derivatives, like tea, wine and honey [4], interact with multiple molecular targets due to their chemical diversity. Their biological activity is determined by their bioavailability, absorption, and metabolism, which are affected by their structure and degree of polymerization. Flavonoids are metabolized into an absorbable aglycone form in the small intestine. However, certain flavonoids resist hydrolytic enzyme activity, which permits their transit to the colon, where they can interact with intestinal cells and the gut microbiota and impact intestinal physiology [5]. Microbial enzymes metabolize flavonoids through glycoside removal, demethylation, and dihydroxylation, generating bioactive low-molecular-weight compounds that possess notable anti-inflammatory and antioxidant properties [6].

**Figure 1:**
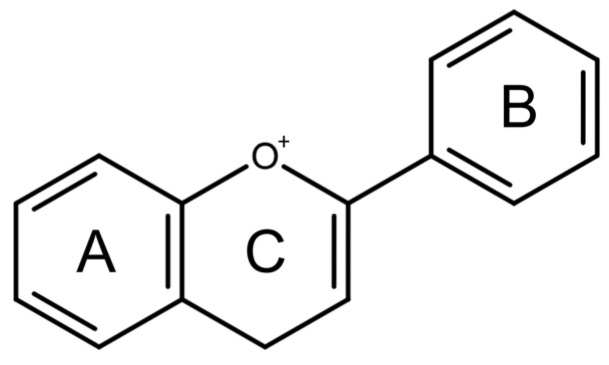
Flavylium cation representing the fundamental structural framework of flavonoids composed of aromatic rings A and B connected by a central oxygen-containing heterocyclic ring C.

Structurally, flavonoids share a flavylium backbone composed of two aromatic rings, A and B, and an aromatic heterocyclic ring, C, collectively identified as the flavylium ion [7] (Figure 1). Flavonoids are classified as anthocyanins, flavonols, flavones, flavanones, isoflavones, or flavan-3-ols on the basis of the number of hydroxyl groups attached to rings A, B, and C (Table 1) [8].

## 3. Colitis, Progression to Colorectal Cancer and Immune Evasion

Inflammatory bowel disease (IBD), including both Crohn’s disease and ulcerative colitis (UC), is characterized by chronic inflammation that affects human health. These conditions are recognized as significant risk factors for developing CAC, with a two- to threefold increased risk compared with that of the general population due to characteristic dysplastic changes [9].

Crohn’s disease can affect any part of the gastrointestinal tract but usually affects the small intestine and/or the colon, with inflammation present across all layers of the bowel wall [3]. In contrast, UC involves nontransmural inflammation confined to the large intestine and rectum. Active UC leads to proinflammatory immune cell infiltration and severe tissue damage, including edema, loss of goblet cells, fibrosis, erosion, and ulcers. Symptoms include diarrhea, abdominal pain, fever, intestinal obstruction, and the passage of blood or mucus [2].

Chronic inflammation is associated with the activation of inflammatory signaling pathways, including the nuclear factor kappa B (NF-κB), IL-6/STAT3, COX-2/PGE2, and IL-23/Th17 pathways [10]. Persistent inflammation in the colon can lead to anatomical changes such as polyps, which may progress from benign lesions to indefinite dysplasia, subsequently to low-grade dysplasia, and eventually to high-grade dysplasia. High-grade dysplasia frequently develops into malignant tumors characterized by uncontrolled cell proliferation, angiogenesis, and immune evasion [11].

Immune cells, including neutrophils, macrophages (Mφs), natural killer (NK) cells, proinflammatory Th1 cells, CD8^+^ lymphocytes, and monocytes, play crucial roles in eliminating transformed cells and reducing the tumor burden at early stages. Nevertheless, if transformed cells escape immune detection, they can progress to later phases of tumor development [12].

**Figure 2:**
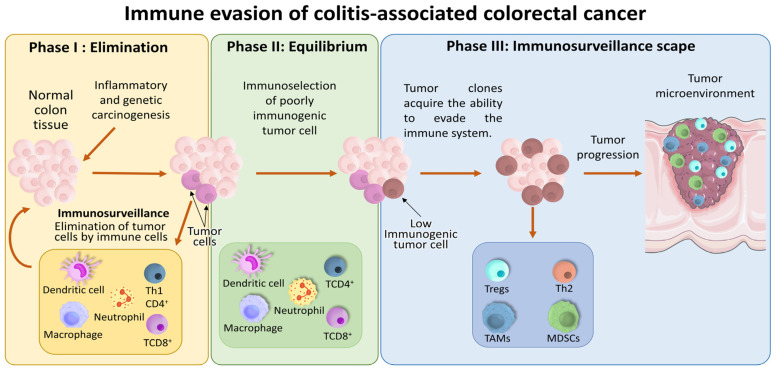
Immunoediting in CAC: Phase I (elimination) immune cells recognize and destroy tumor cells (immunosurveillance). In phase II (equilibrium), tumor cell death matches their generation, promoting immunoselection of poorly immunogenic clones. In phase III (escape), tumor cells evade the immune response and proliferate, establishing an immunosuppressive tumor microenvironment dominated by regulatory T cells (Tregs), tumor-associated macrophages (TAMs), myeloid suppressor cells (MDSCs), macrophages (Mφs) and type 2 helper T (Th2) cells. The orange arrows illustrate the cellular transitions through immune evasion.

During the second phase, immunogenicly transformed cells are eliminated, while those not recognized by the immune system persist and promote tumor growth, leading to immunoselection [13]. In the third phase, there is a shift toward an anti-inflammatory immune response mediated by Th2 cells, regulatory T cells (Tregs), and tumor-associated immune cells with permissive profiles, such as tumor-associated macrophages (TAMs) and myeloid-derived suppressor cells (MDSCs), which facilitate tumor progression and angiogenesis necessary for metastasis [14]. Tregs promote an anti-inflammatory environment by secreting IL-10 and TGF-β, both of which are linked to poor CRC prognosis [15] (Figure 2).

The mechanisms described are dynamic, shifting from an inflammation-associated environment during CRC initiation to a predominantly anti-inflammatory context that supports tumor growth and metastasis. Flavonoids with anti-inflammatory properties may mitigate colitis and play protective roles against CAC progression. However, once CAC is established, their effects require careful evaluation, as outcomes may depend on the balance of signaling cues in the tumor microenvironment and the specific stage of CRC development.

## 4. Mechanisms of Action of Flavonoids

Both in vivo and in vitro studies have demonstrated that flavonoids effectively reduce inflammation in the digestive tract at doses ranging from 10 to 200 mg/kg body weight. These effects are mediated via various mechanisms, including the following:

### 4.1. Modulation of the Microbiota by Flavonoids

Intestinal dysbiosis is strongly linked to gastrointestinal tract inflammation and plays a significant role in the development of CAC [16]. Pathobionts have also been shown to promote an immunosuppressive tumor microenvironment that favors the growth of CRC. In addition, both clinical and preclinical evidence indicate that bacteria can colonize tumors [17,18,19]. For example, *F. nucleatum* is consistently enriched in the intratumoral microbiome of patients with CRC and can persist in metastatic lesions [20,21].

The microbial shifts observed during the transition from inflammation to malignancy are detailed in Table 2. These specific taxonomic changes represent targets for intervention with flavonoids.

Flavonoids contribute to the enhancement of the composition and functionality of the intestinal microbiota by promoting beneficial bacteria, although the effects of these compounds vary. For example, hesperetin and myricetin have been shown to increase the abundance of beneficial genera such as *Bifidobacterium* and *A. muciniphila* while reducing potentially pathogenic groups such as *Enterobacteriaceae*, all of which are linked to maintaining intestinal barrier integrity and mitigating inflammation [22], a key mechanism in their protective role against CAC progression. Additionally, flavonoids like quercetin and luteolin help reduce the production of procarcinogenic metabolites by pathogenic bacteria such as *F. nucleatum*, *P. anaerobius*, *E. coli*, and *C. difficile* in patients with colitis [23,24] (see Table 3 for details).

However, studies are needed to confirm whether in advanced stages of CAC, flavonoids can decrease the intratumoral microbiome and the degree of immunosuppression in the tumor microenvironment by regulating these pathogenic bacteria.

**Table 2:** Microbiota shifts and dysbiosis during colorectal inflammation and cancer progression. ↑ = increase.

Condition	Microbial Changes (Key Taxa)	Function	Ref.
Healthy gut microbiota	*Roseburia hominis*, *Dorea formicigenerans*, and *Ruminococcus obeum*	Symbiotic and anti-inflammatory species.	[23,25]
Colon inflammation (Ulcerative colitis)	↑ *Bifidobacterium breve* and *Clostridium* *symbiosum.*	Inflammatory dysbiosis.	
	↑ Hydrogen sulfide (H_2_S), Ammonia (NH_3_)	Genotoxic and proinflammatory.	
(Crohn’s disease)	↑ *Ruminococcus gnavus, Escherichia coli*, *Clostridium clostridioforme*	Inflammatory dysbiosis	
Colorectal adenoma	↑ *Fusobacterium nucleatum*, *Bacteroides fragilis (ETBF)*, *Solobacterium moorei*, *Escherichia coli*, *Lachnoclostridium* spp., *Peptostreptococcus anaerobius*, *E. faecalis*, *Parvimonas micra*	Tumor-promoting bacteria enriched in adenomas.	[26,27,28]
Colorectal cancer	↑ *Morganella morganii*, *Clostridium butyricum, (ETBF)*, *S. thermophilus*, *S. gallolyticus*, *S. salivarius*, *L. gallinarum*, *S. moorei*, *P. stomatis*, *P. asaccharolytica*, *P. intermedia*, *Carnobacterium maltaromaticum*, pks + *E. coli*	Strong immunosuppressive/ procarcinogenic signals.	[29,30,31,32,33,34,35]
	↑ *B. fragilis (enterotoxogenic)*	Disrupts intestinal barrier, activates Th17, STAT3, NF-κB, suppresses exosomal miR-149-3p, promotes Th17 differentiation.	[33,36,37]
	↑ *P. micra*	Stimulates Th17 cell responses, proinflammatory cytokines	[38]
	↑ *F. nucleatum*, *P. anaerobius*, *E. faecalis*	Activate TLR2/4 signaling via MYD88, trigger NF-κB, promote cytokines, tumor progression and metastasis.	[39,40,41]
	↑ *F. nucleatum*	Local enrichment in intratumoral microbiome, in metastatic lesions.	[20,21]
	↑ *P. anaerobius*	Promote MDSC infiltration by inducing CXCL1 (intratumoral).	[40,42]
	↑ *Klebsiella pneumoniae*	Regulate tumor cytokines, attract M2 Mφs, suppress T-cell functions (intratumoral).	[43]

### 4.2. Flavonoids Regulate the Expression of Tight Junction Proteins in the Gut Epithelial Barrier

**Figure 3:**
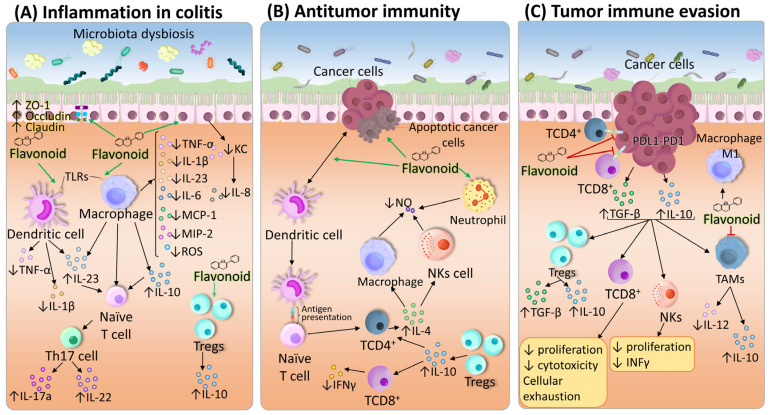
Immunomodulatory effects of flavonoids during colitis and tumorigenesis. (**A**) Regulation of tight junctions, cytokines, reactive oxygen species (ROS) and cells during colitis. (**B**) Flavonoid effects on cytokines, ROS and cells involved in antitumor immunity. (**C**) Influence on cytokines and cells involved in immune evasion of colon cancer cells. ↑ indicates upregulation, and ↓ indicates downregulation by flavonoids on the indicated biomolecule or cell. The green arrows indicate the activation or induction of biomolecules or cells by flavonoids, whereas the red lines denote flavonoid-mediated inhibition of specific interactions or cellular functions. The black arrows illustrate the endogenous biological pathways, cellular transitions, and signaling cascades within the colonic and tumor microenvironments.

Individuals genetically predisposed to UC exhibit a compromised epithelial barrier, characterized by structural abnormalities in tight junctions [44]. This barrier dysfunction permits luminal antigens to penetrate the intestinal mucosa, promoting the infiltration of Mφs, dendritic cells (DCs), neutrophils, and other immune cells [24]. The resulting chronic inflammatory environment causes tissue damage and contributes to UC pathogenesis [3]. Notably, flavonoids such as quercetin, rutin and hesperetin, among others, enhance the expression of the key membrane-associated proteins Zonula occludens-1 (ZO-1), occludin, and claudin-1, reinforcing epithelial integrity within the colon (see Table 3 for details; Figure 3A).

**Table 3:** Flavonoid immunomodulatory effects. ↑ indicates positive and ↓ negative regulation of flavonoids on the mentioned effect.

Flavonoid	Model	Dose	Immunomodulatory Effects	Ref.
Acacetin	RAW264.7 cells	45 μmol/L 24 h	↓ TNF-α, IL-1β, IL-6, IL-23, IFN-γ, IL-12, IL-8, IL-17, IL-18 and IL-33, ↓ iNOS, ↓ COX-2 and PGE2	[45]
	C57BL/6 mouse model of dextran sulfate sodium (DSS)-induced colitis	50 mg/kg by oral gavage (p.o.) from day 1 to day 9		
Anthocyanins	Caco-2, HT-29, HCT-116 cells	200 μmol/L, 400 μg/mL, 154.3 μg/mL for 48 h	↓ MIP-1, ↓ MAPK (p38, ERK1/2, JNK), ↓ COX-2 and PGE2. Useful in the treatment of cancer	[46,47,48]
Apigenin	C57BL/6 mouse model of DSS-induced colitis	25 and 50 mg/kg p.o. for 3 days	↑ Beneficial bacteria, ↑ ZO-1, occludin and claudin-1, ↓ TNF-α, IL-1β, IL-6, IL-23, IFN-γ, IL-12, IL-8, IL-17, IL-18 and IL-33, ↑ IL-10 and TGF, ↓ MAPK (p38, ERK1/2, JNK), ↓ MPO, ↓ iNOS, ↓ COX-2 and PGE2	[49,50,51]
	Caco-2 cells C57BL/6 mouse model of oxazolone-induced colitis	50 μM 1, 12 and 24 h 0.8 mg/day from day 0 to day 35		
Baicalein	BALBc mouse model of DSS-induced colitis	40 mg/kg p.o. daily for 10 days beginning with the start of DSS exposure	↓ STAT3 and/or STAT6	[52]
Baicalin	Sprague–Dawley (SD) rat model of 2,4,6-trinitrobenzenesulfonic acid (TNBS)-induced colitis, HT-29 cells	100 mg/kg p.o. once a day for 14 days, 316 µg/mL for 24 h	↓ TNF-α, IL-1β, IL-6, IL-23, IFN-γ, IL-12, IL-8, IL-17, IL-18 and IL-33, ↓ NF-κB, ↓ MIP-3, ↑ Tregs, ↓ MDA, ↑ GPx and CAT. Useful in the treatment of cancer	[53,54,55,56,57]
	Peripheral blood mononuclear cells from patients with UC	40 μmol/L for 24 h		
	SD rat model of TNBS-induced colitis, RAW264.7 cells	5 mg/mL p.o. per day, 5.0 × 10^−5^ μM + 1 μg/mL LPS for 48 h		
	SD rat model of TNBS-induced colitis	Intragastrically (i.g.) 1% (*w*/*v*) once every 2 days for 14 days		
	SD rat model of TNBS-induced colitis	i.g. 90 mg/kg daily through the model		
Chrysin	BALBc mouse model of DSS-induced colitis	10 mg/kg p.o. for 7 days, beginning with the start of DSS exposure.	↓ TNF-α, IL-1β, IL-6, IL-23, IFN-γ, IL-12, IL-8, IL-17, IL-18 and IL-33, ↓ MIP-1, ↓ MPO	[58]
Daidzein	Mesenteric lymph node cells from DSS-induced C57BL/6 mouse model of DSS-induced colitis	100 mg/kg daily from day −7 to 6 of DSS model	↓ TNF-α, IL-1β, IL-6, IL-23, IFN-γ, IL-12, IL-8, IL-17, IL-18 and IL-33, ↓ NF-κB, ↓ TLR4, ↑ IL-10 and TGF, ↓ MPO, useful in the treatment of cancer	[59,60]
	BALBc mouse model of DSS-induced colitis, RAW 264.7 cells	10 mg/kg p.o. for seven days, 200 μM/mL for 24 h		
EGCG	Caco-2 cells	5 μM for 6 h	↑ Beneficial bacteria, ↑ ZO-1, occludin and claudin-1, ↓ TNF-α, IL-1β, IL-6, IL-23, IFN-γ, IL-12, IL-8, IL-17, IL-18 and IL-33, ↓ NF-κB, ↓ TLR4, ↓ MIP-3, ↓ MIP-1, ↑ Tregs, ↑ IL-10 and TGF, ↓ STAT3 and/or STAT6, ↓ MPO, useful in the treatment of cancer	[61,62,63,64,65,66,67]
	HT29 and T84 cells	25 μM for 24 h		
	C57BL/6 mouse model of DSS-induced colitis	100 mg/kg p.o. through DSS model		
Epicatechin	C57BL/6 mouse model of DSS-induced colitis, Caco-2 cells	300 mg/kg p.o. from the beginning to the end of the model, 5 μM incubated for 6 h	↑ ZO-1, occludin and claudin-1, ↓ TNF-α, IL-1β, IL-6, IL-23, IFN-γ, IL-12, IL-8, IL-17, IL-18 and IL-33, ↓ NF-κB, ↓ TLR4, ↓ MIP-3, ↓ MIP-1, ↑ Tregs, ↑ IL-10 and TGF, ↓ STAT3 and/or STAT6, ↓ MPO, ↓ GSH and SOD, ↑ GPx and CAT. Useful in the treatment of cancer	[62,68]
	BALBc mouse model of DSS-induced colitis	100 mg/kg/day p.o. on day 8 of the experiment		
	C57BL/6 mouse model of DSS-induced colitis	50 mg/kg from day 5 to day 12		
	Specific-pathogen-free SD rat model of TNBS-induced colitis	50 mg/kg/d i.p. for 10 consecutive days after model establishment		
	C57BL/6 model of TNBS-induced colitis	10 mg/kg i.p. twice a day after the induction of colitis		
Eriocitrin	C57BL/6J mouse model of DSS-induced colitis	30 mg/kg/d p.o. followed by DSS administration	↓ TNF-α, IL-1β, IL-6, IL-23, IFN-γ, IL-12, IL-8, IL-17, IL-18 and IL-33, ↓ MPO	[69]
Fisetin	BALBc mouse model of DSS-induced colitis, BALBc peritoneal Mφs	10 mg/kg p.o. once a day from one day before of DSS administration and followed until the 8th day, 50 μM for 24 h	↓ TNF-α, IL-1β, IL-6, IL-23, IFN-γ, IL-12, IL-8, IL-17, IL-18 and IL-33, ↓ NF-κB, ↓ MAPK (p38, ERK1/2, JNK), ↓ MPO, ↓ iNOS, ↓ GSH and SOD, ↓ MDA, ↓ COX-2 and PGE2. Useful in the treatment of cancer	[70]
Galangin	Swiss albino mouse model of DSS-induced colitis	40 mg/kg/d p.o. from day 8 to day 28	↓ TNF-α, IL-1β, IL-6, IL-23, IFN-γ, IL-12, IL-8, IL-17, IL-18 and IL-33, ↓ NF-κB, ↓ TLR4, ↑ IL-10 and TGF, ↓ MPO, ↓ iNOS, ↓ GSH and SOD, ↓ COX-2 and PGE2. Useful in the treatment of cancer	[71,72,73,74]
	RAW 264.7 cells	Pretreated with 0.78 μg/mL for 1 h		
	BALBc mouse model of DSS-induced colitis	40 mg/kg p.o. from day 0 to 12		
	ICR mouse model of DSS-induced colitis	15 mg/kg p.o. from 1 week prior to the DSS challenge, up to the end of study		
Genistein	Wister rat model of acetic acid (AA)-induced colitis	100 mg/kg/d equivalent to a human dose of 16.13 mg/kg	↑ Beneficial bacteria, ↑ ZO-1, occludin and claudin-1, ↓ TNF-α, IL-1β, IL-6, IL-23, IFN-γ, IL-12, IL-8, IL-17, IL-18 and IL-33, ↓ MIP-1, ↓ STAT3 and/or STAT6, ↓ MPO, ↓ iNOS, ↓ COX-2 and PGE2. Useful in the treatment of cancer	[75,76,77,78,79]
	C57BL/6 mouse model of DSS-induced colitis	10 mg/kg/d p.o. till day 14th		
	C57BL/6 mouse model of DSS-induced colitis, THP-1 cells and U937 cells	45 mg/kg from day 1 to day 10, 20 μM for 4 h		
	Wistar rat model of TNBS-induced colitis	100 mg/kg p.o. 24 h after TNBS administration for 14 days		
	C57BL/6 mouse model of DSS-induced colitis	40 mg/kg/d i.g. for 10 days		
Hesperetin	C57BL/6 mouse model of DSS-induced colitis, Caco-2 cells	40 mg/kg/d p.o. from day 0 to day 13, 40 µg/mL for 48 h	↑ ZO-1, occludin and claudin-1, ↓ TNF-α, IL-1β, IL-6, IL-23, IFN-γ, IL-12, IL-8, IL-17, IL-18 and IL-33, ↓ MIP-1, ↑ Tregs, ↑ IL-10 and TGF, ↓ MPO, ↓ GSH and SOD, ↓ MDA	[76,80,81,82,83]
	C57BL/6 mouse model of DSS-induced colitis, Caco-2 and RAW264.7 cells	20 mg/kg/d i.p., 100 μM for 24 h		
	Wistar rat model of DSS-induced colitis	50 mg/kg/d p.o. for 14 days		
	BALB/c mouse model of DSS-induced colitis	80 mg/kg/d p.o. at the same time as DSS		
Kaempferol	C57BL/6J mouse model of DSS-induced colitis	0.3% diets for 3 weeks after the start of DSS exposure	↑ Beneficial bacteria, ↓ TNF-α, IL-1β, IL-6, IL-23, IFN-γ, IL-12, IL-8, IL-17, IL-18 and IL-33, ↓ MPO, ↓ iNOS, ↓ COX-2 and PGE2	[61,84]
Linarin	C57BL/6J mouse model of DSS-induced colitis	50 mg/kg/d p.o. beginning with the start of DSS exposure	↑ Beneficial bacteria, ↓ TNF-α, IL-1β, IL-6, IL-23, IFN-γ, IL-12, IL-8, IL-17, IL-18 and IL-33, ↓ MPO	[85]
Luteolin	Caco-2 cells	150 μM for 24 h	↑ Beneficial bacteria, ↓ TNF-α, IL-1β, IL-6, IL-23, IFN-γ, IL-12, IL-8, IL-17, IL-18 and IL-33, ↓ NF-κB, ↓ MAPK (p38, ERK1/2, JNK), ↓ STAT3 and/or STAT6, ↓ MPO, ↓ iNOS, ↓ GSH and SOD, ↓ MDA, ↓ COX-2 and PGE2. Useful in the treatment of cancer	[73,86,87,88,89,90]
	C57BL/6CrSlc mouse model of DSS-induced colitis, Caco-2, L929, and RAW 264.7	50 mg/kg/d p.o. starting 7 days before DSS treatment and continuing until sacrifice, 100 μM after 6 h		
	C57BL/6 mouse model of DSS-induced colitis	100 mg/kg/d p.o. after DSS administration from days 3–7		
	C57BL/6 mouse model of DSS-induced colitis	50 mg/kg/d p.o. 7 days prior to DSS exposure and were then maintained until sacrifice		
	SPF grade C57BL 6 mouse model of DSS-induced colitis	20 mg/kg i.p. after DSS administration		
Myricetin	C57BL/6 mouse model of DSS-induced colitis	80 mg/kg p.o. at the same time as DSS	↓ TNF-α, IL-1β, IL-6, IL-23, IFN-γ, IL-12, IL-8, IL-17, IL-18 and IL-33, ↓ NF-κB, ↑ Tregs, ↑ IL-10 and TGF, ↓ MPO, ↓ GSH and SOD, ↓ MDA, ↑ GPx and CAT	[91,92,93]
	pathogen-free BALB/c mouse model of DSS-induced colitis	200 mg/kg/d p.o. beginning on the day on which oral DSS water was given		
	CD1 mouse model of DSS-induced colitis	10 mg/kg/d p.o.; the treatment started 1 h before administration of DSS and followed until the seventh day		
Myricitrin	CD1 mouse model of DSS-induced colitis	10 mg/kg/d p.o. for 7 days beginning with the start of DSS exposure	↓ TNF-α, IL-1β, IL-6, IL-23, IFN-γ, IL-12, IL-8, IL-17, IL-18 and IL-33, ↓ MAPK (p38, ERK1/2, JNK), ↓ NF-κB, ↓ COX-2 and PGE2. Useful in the treatment of cancer	[93]
Naringenin	RAW264.7 cells and HT-29 cells, C57BL/6 mouse model of DSS-induced colitis	25 µmol/L for 2 h, 50 mg/kg p.o. prior to DSS treatment and continued till the end of DSS treatment	↑ Beneficial bacteria, ↑ ZO-1, occludin and claudin-1, ↓ TNF-α, IL-1β, IL-6, IL-23, IFN-γ, IL-12, IL-8, IL-17, IL-18 and IL-33, ↓ NF-κB, ↓ TLR4, ↓ MIP-1, ↓ iNOS, ↓ GSH and SOD, ↑ GPx and CAT, ↓ COX-2 and PGE2. Useful in the treatment of cancer	[94,95,96,97,98]
	BALB/c mouse model of DSS-induced colitis	Diet containing 0.3% (*w*/*w*) during the recovery period (11 days)		
	Wistar rat model of AA-induced colitis	100 mg/kg/d p.o. for 7 consecutive days pre colitis induction		
	BALB/c mouse model of DSS-induced colitis	diet containing 0.3% (wt:wt) till the end of DSS treatment		
Naringin	C57BL/6 mouse model of DSS-induced colitis	100 mg/kg p.o. the same days as DSS treatment	↑ Beneficial bacteria, ↑ ZO-1, occludin and claudin-1, ↓ NF-κB, ↓ MAPK (p38, ERK1/2, JNK), ↓ MPO, ↓ iNOS, ↓ GSH and SOD, ↓ MDA, ↓ COX-2 and PGE2. Useful in the treatment of cancer	[99,100,101]
	Wistar rat model of AA-induced colitis	80 mg/kg p.o. for 12 days		
	C57BL/6 mouse model of AA-induced colitis, RAW264.7 cells and rat intestinal IEC-6 epithelial cells	40 mg/kg/d p.o. for seven successive days, 1 day after colitis induction, 20 μM in vitro for 12 h		
Pinocembrin	RAW264.7 and Caco-2 cells, C57BL/6 mouse model of DSS-induced colitis	150 μM for 24 h, 100 mg/kg/d p.o. 2 days prior to DSS treatment and continued to the end of the DSS treatment	↑ ZO-1, occludin and claudin-1, ↓ TNF-α, IL-1β, IL-6, IL-23, IFN-γ, IL-12, IL-8, IL-17, IL-18 and IL-33, ↓ NF-κB, ↓ TLR4, ↓ iNOS, ↓ COX-2 and PGE2. Useful in the treatment of cancer	[102,103]
	SD rat model of DSS-induced colitis	50 mg/kg p.o. for 7 days, prior to the DSS treatment and treated until the last day of the experiment		
Quercetin	Fischer rat model of DSS/azoximetane (AOM)-induced CAC	25 mg/kg injected 3 days a week during the 18 weeks of the experiment	↑ Beneficial bacteria, ↑ ZO-1, occludin and claudin-1, ↓ TNF-α, IL-1β, IL-6, IL-23, IFN-γ, IL-12, IL-8, IL-17, IL-18 and IL-33, ↓ NF-κB, ↓ TLR4, ↑ Tregs, ↑ IL-10 and TGF, ↓ MAPK (p38, ERK1/2, JNK), ↓ STAT3 and/or STAT6, ↓ MPO, ↓ iNOS, ↓ GSH and SOD, ↑ GPx and CAT, ↓ COX-2 and PGE2. Useful in the treatment of cancer	[104,105,106,107,108,109,110]
	C57BL/6 mouse model of DSS-induced colitis and *Ahr^−^^/^^−^* mouse model	50 mg/kg p.o. from days 1–10 and 18–22		
	RAW264.7 cell	10 μM for 24 h		
	Swiss rat model of AA-induced colitis	100 mg/kg p.o.; the animals were treated 2 h before and 10 h after colitis induction, and they were euthanized at the 18th hour		
	Rat intestinal microvascular endothelial cells	80 μM for 12 h		
	Wistar rat model of TNBS-induced colitis, Caco-2 cells,	25 mg/kg/d, p.o. for 11 days, 493 μM for 2 h		
	C57BL/6 mouse model of DSS-induced colitis	Mice chow pellets supplemented with 100, 500, 1000, and 1500 ppm quercetin. After one week, mice quercetin with 3% (m/v) DSS added for 6 days		
Rutin	BALBc mouse model of DSS-induced colitis	50 mg/kg/d for 48 days beginning with the start of DSS exposure,	↑ ZO-1, occludin and claudin-1, ↓ TNF-α, IL-1β, IL-6, IL-23, IFN-γ, IL-12, IL-8, IL-17, IL-18 and IL-33, ↓ NF-κB, ↓ MAPK (p38, ERK1/2, JNK), ↓ MPO, ↓ iNOS	[111,112]
	pathogen-free ICR mouse model of DSS-induced colitis, peritoneal Mφs	6 mg/kg/d, p.o. for 2 weeks 1-week DSS exposure, 200 μM for 30 min		
Tangeretin	C57BL/6 mouse model of TNBS-induced colitis	20 mg/kg/d p.o. for 3 days after treatment with TNBS	↑ ZO-1, occludin and claudin-1, ↓ TNF-α, IL-1β, IL-6, IL-23, IFN-γ, IL-12, IL-8, IL-17, IL-18 and IL-33, ↓ NF-κB, ↑ Tregs, ↑ IL-10 and TGF, ↓ MAPK (p38, ERK1/2, JNK), ↓ MPO. Useful in the treatment of cancer	[50,113,114]

### 4.3. Negative Regulation of Proinflammatory Cytokines and Chemokines by Flavonoids

Mφs and DCs infiltrating the colonic tissue recognize microbiota-associated molecular patterns and sample luminal antigens via their Toll-like receptors (TLRs) and C-type lectin receptors (CLRs) [115]. Throughout the progression of UC, the immune response promotes persistent inflammation, characterized by elevated levels of reactive oxygen species (ROS), proinflammatory cytokines, and chemokines. These factors collectively induce DNA damage and trigger oncogenic signaling pathways [116].

Antigen recognition by Mφs and their subsequent interaction with T lymphocytes drive the maturation of T cells into effector cells. The production of proinflammatory cytokines, such as TNF-α, IL-1β, IL-6, IL-23, and IL-17a, amplifies inflammation by recruiting and activating additional mediators such as IFN-γ and IL-22 [117]. Chemokines, including MCP-1, IL-8, and MIP-2, facilitate the recruitment and infiltration of leukocyte populations that contribute to tissue damage [118].

Flavonoids have been shown to inhibit the secretion of TNF-α, IL-1β, IL-6, IL-23, IFN-γ and IL-12 and, to a lesser extent, of IL-8, IL-17, IL-18 and IL-33 [24] and decrease the expression of TLR4, MIP-3 and MCP-1, helping reduce chronic intestinal inflammation. For example, luteolin has been shown to inhibit TLR4 and downstream NF-κB activation, thereby reducing the secretion of TNF-α, IL-6, and IL-1β in murine models of colitis. Similarly, baicalein attenuates intestinal inflammation by suppressing TLR4-mediated pathways and decreasing the levels of chemokines such as CCL2 and CXCL1. These actions contribute to the resolution of chronic inflammation and the restoration of intestinal homeostasis, underscoring their therapeutic potential during the early stages of CAC. The detailed molecular targets and immune effects of several dietary polyphenols are summarized in Table 3 (Figure 3A).

In oncological contexts, when Mφs are stimulated with IFN-γ and TLR ligands, they can mediate tumor cell lysis [119]. Therefore, flavonoids that decrease IFN-γ or downregulate TLR expression while broadly suppressing proinflammatory cytokines and chemokines may, under certain conditions, impair tumor cell elimination. For example, luteolin is a well-characterized NF-κB inhibitor that decreases TNF-α, IL-6, and IL-1β expression, which are key for driving effector T-cell recruitment and macrophage activation [120]. Persistent suppression of these inflammatory signals, especially in advanced CAC, may limit antitumor immunity and contribute to immune evasion.

This effect is not generalizable to all contexts but rather depends strongly on the chemical structure of flavonoids, their concentration, and the stage of disease. For instance, structurally, planar flavonoids such as baicalein bind to the aryl hydrocarbon receptor (AhR), influencing transcriptional programs that include immunoregulatory cytokines and Treg-related genes [121]. At high doses, some AhR ligands have been linked to increased Treg expansion and IL-10 production, both of which are associated with the suppression of effector T-cell function in tumors [122].

Thus, while the anti-inflammatory activity of flavonoids is clearly beneficial during early colitis, in more advanced stages of CAC, when a type 1 immune response is required for tumor control, their prolonged or high-dose use may shift the balance toward immunosuppression. This observation is further supported by reports that some flavonoids increase the proportion of Tregs, which suppress CD8^+^ T-cell activity and facilitate tumor escape during the immune equilibrium and escape phases of CAC [123] (Figure 3B).

In the third phase of immune evasion, tumor cells, along with Tregs, contribute to an anti-inflammatory profile by secreting increased amounts of IL-10 and TGF-β. This environment supports additional IL-10 secretion by monocytes and Mφs and suppresses NK cell-mediated cytotoxicity within the tumor microenvironment. Elevated serum IL-10 levels are associated with a poorer prognosis regarding treatment response and disease-free survival in patients with CRC [15], whereas TGF-β is involved in cancer cell growth, invasion, and metastasis and reduces antitumor immune responses by inhibiting the differentiation, maturation, and proliferation of CD8^+^ T lymphocytes, as well as the maturation and presentation of tumor antigens by DCs [124] (Figure 3C). Flavonoids such as apigenin, luteolin or naringenin have been shown to induce the secretion of IL-10, TGF-β, or both, as well as promote Treg differentiation (Table 3). The mechanisms underlying the effects of flavonoids on cytokine production are still being studied; however, flavonoid-mediated effects on cytokine secretion and Treg populations contribute to the resolution of chronic inflammation and may play a role in modulating immune responses during colitis-associated with colorectal cancer progression; detailed targets and immune effects are summarized in Table 3.

#### 4.3.1. Flavonoid Inhibition of the NF-κB Signaling Pathway

NF-κB is a family of transcription factors that controls inflammatory responses through the regulation of chemokines and proinflammatory cytokine gene expression. These factors are involved in cell proliferation and death processes, which are related to oncogenesis by regulating the expression of various cell cycle controllers, such as cyclin A, cyclin D1, and cyclin-dependent kinase 6 (CDK6) [125]. In some instances, cancer cells display concurrent NF-κB activation and unregulated proliferation or reduced sensitivity to cell death [126]. Notably, NF-κB also plays a key role in antitumor immunity by activating antigen-presenting cells and promoting the proinflammatory cytokines essential for defense. Therefore, total and systemic inhibition of NF-κB can be detrimental, as this transcription factor is essential for immune activation and the maturation of antigen-presenting cells, and it controls the expression of numerous proinflammatory cytokines and chemokines that are key for recruiting and activating effector immune cells such as Mφs and T cells [127]. Solely focusing on broad NF-κB suppression may unintentionally dampen these critical signals, which could hinder immune surveillance and facilitate tumor evasion. In contrast, context-dependent modulation that limits protumorigenic NF-κB signaling in cancer cells while maintaining its activity in effector immune cells may provide therapeutic benefit.

**Figure 4:**
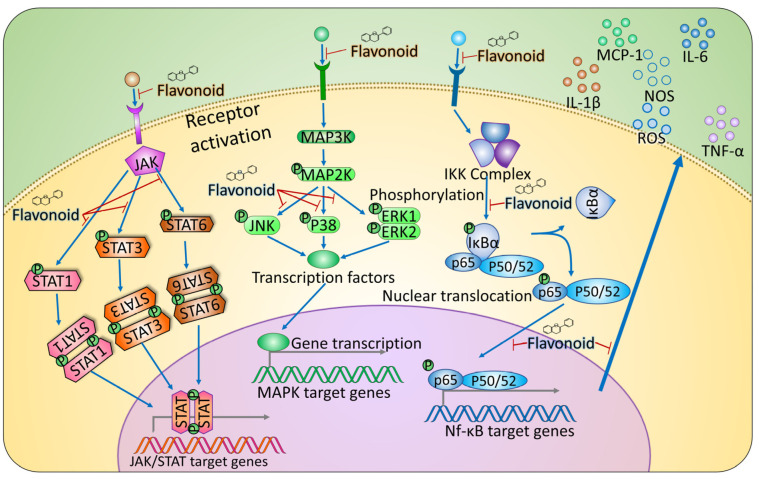
Flavonoid-mediated inhibition of the JAK/STAT, MAPK and NF-κB signaling pathways. The blue arrows illustrate the signaling cascades while red lines denote flavonoid-mediated inhibition of specific interactions.

Flavonoids regulate NF-κB signaling at multiple levels of the cascade. For example, baicalein and quercetin, among other dietary flavonoids, have been shown to directly inhibit key steps of NF-κB activation, including the phosphorylation and degradation of IκBα and the nuclear translocation of the p65 subunit, leading to reduced expression of proinflammatory mediators in experimental models of inflammation [128,129] (Table 3; Figure 4). However, such multilevel modulation must be interpreted with care, as the impacts on immune cells and cancer cells may differ. In immune effector cells like CD8^+^ T lymphocytes and NK cells, excessive inhibition of NF-κB signaling can reduce the production of IFN-γ and other effector cytokines that support antitumor immunity, which in turn may favor a microenvironment permissive to tumor growth [130].

These observations emphasize that the effect of NF-κB modulation by flavonoids is not uniform across all cell types or disease stages. A fine-tuned approach that preserves NF-κB activity, where it supports protective inflammation and immune activation while selectively attenuating its protumorigenic signaling in transformed or stromal cells, is essential.

#### 4.3.2. Flavonoids Inhibit the Mitogen-Activated Protein Kinase (MAPK) Pathway

The MAPK pathway, which involves the ERK1/2, JNK, and p38 kinases, is a key regulator of various cellular processes, including proliferation, differentiation, migration, and apoptosis [131].

The pivotal ERK1/2 signaling axis controls cell growth, proliferation, differentiation, and apoptosis. Furthermore, it has roles in inflammation and CAC, promoting tumor cell survival and proliferation while also contributing to antitumor inflammatory responses. Early activation of ERK1/2 can restrict initial tumor development but may facilitate metastasis during advanced disease stages [132]. This complexity underscores the necessity of considering the disease stage prior to the use of flavonoids, which downregulate this pathway, and the stage of development of this disease should be considered (Table 3).

The JNK pathway modulates cellular responses to stress, inflammation, and apoptosis and can be activated by irradiation, environmental stressors, and growth factors. In contrast, p38 MAPKs are notably involved in autoimmune mechanisms, regulating the production of TNF-α, IL-1β, and IL-6. Stimulation of the p38 pathway by IL-1β, TNF-α, TGF-β or oxidative stress [133] can establish a feedback loop amplifying inflammation and potentially suppressing tumorigenesis via transcriptional and posttranscriptional regulation at early transformation stages. However, following the acquisition of a malignant phenotype, p38 activation may enhance tumor cell growth [134]. Given the dual role of this pathway, careful assessment of disease progression is needed before flavonoids that attenuate its activation, such as apigenin and luteolin, can be used. The detailed effects of several dietary polyphenols on the MAPK pathway are summarized in Table 3 (Figure 4). For individuals with UC without neoplastic transformation, p38 inhibition may help prevent chronic inflammation. Conversely, in those with precancerous lesions, reduced p38 activity could hinder the elimination of transformed cells. In advanced cancer stages, targeted modulation of the p38 pathway may be advantageous for controlling malignant tumor progression.

#### 4.3.3. Flavonoids Inhibit the Signal Transducer and Activator of Transcription (STAT) Activation

STAT proteins are a family of transcription factors that regulate gene expression, influencing cell differentiation, proliferation, survival, and inflammation in response to cytokines and growth factors via JAK-mediated tyrosine phosphorylation [135].

The relationship between STATs and cytokines involves a complex and dynamic feedback system. Cytokines activate these pathways and promote the expression of more cytokines, which are influenced by cell type, stimulus and the cellular microenvironment [136]. For example, STAT3 and STAT6 play crucial roles in the connection between inflammation and the development of CAC. In contrast, STAT1 may function as a tumor suppressor in inflammation-linked carcinogenesis [137].

STAT3 is predominantly activated by IL-6, IL-10, IL-35, and epidermal growth factor (EGF) [138]. In particular, IL-6 signaling drives proinflammatory gene expression and fosters carcinogenesis by inducing genes involved in angiogenesis, invasion, and metastasis [139].

STAT6 activation occurs primarily through IL-4 and IL-13, facilitating Mφ differentiation toward the M2 phenotype, modulating T-cell proliferation and survival, suppressing CD8^+^ T-cell effector functions, promoting Th2 differentiation, and regulating Treg activity [140].

STAT1 is mainly activated via IFN-γ, leading to the expression of crucial genes for immune defense against pathogens and tumors, including CXC motif chemokine ligands (CXCLs) and microRNAs [141]. Furthermore, STAT1 upregulates proapoptotic genes essential for TNF-mediated apoptosis [142].

Chronic activation of the STAT3 and STAT6 pathways is correlated with persistent inflammation and oncogenesis. Flavonoids have been investigated as potential STAT inhibitors; however, owing to the intricate regulation of STAT proteins and their interactions within the cellular microenvironment, nonselective inhibition could lead to unfavorable outcomes. Specifically, flavonoids that inhibit STAT3 and/or STAT6 signaling might attenuate inflammatory processes and block tumor-promoting pathways. Among these, luteolin stands out as a well-characterized compound capable of inhibiting STAT3 and, in certain inflammatory contexts, STAT6 activation, thereby limiting protumorigenic and Th2 immune responses. Similar inhibitory effects on STAT3 have been described for baicalein and EGCG, although their mechanisms and specificity vary depending on the cellular context and disease stage (Table 3; Figure 4). While the desired therapeutic goal is the inhibition of protumorigenic and proinflammatory signaling mediated by STAT3 and STAT6, genistein can paradoxically interfere with the activation of STAT1, which is essential for the induction of IFN-γ-dependent genes and facilitates tumor surveillance and the cytotoxic activity of immune cells. The mechanism behind this opposite effect involves the ability of the genistein molecule to compete for the ATP-binding site of Janus kinases (JAKs), where at certain concentrations, it may have a greater affinity for the pathways that lead to STAT1 phosphorylation than for those that lead to STAT3 phosphorylation [143]. Consequently, the use of genistein might impair the primary defense mechanisms against malignancy by dampening STAT1-mediated antitumor immunity, even while attempting to reduce chronic inflammation.

### 4.4. Flavonoid Protection Against Oxidative Stress

**Figure 5:**
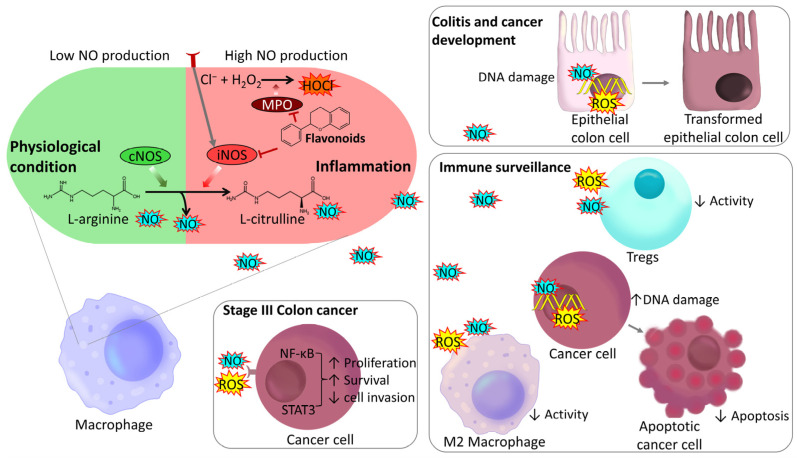
Modulation of oxidative stress by flavonoids and the role of ROS and NO in different stages of colitis and cancer development. Reduction in the level of ROS/RNS by direct antioxidant activity via the inhibition of iNOS and MPO. ↑ indicates upregulation, and ↓ indicates downregulation by flavonoids on the indicated cell or biological process. The grey arrows illustrate the endogenous biological pathways, cellular transitions and signaling cascades. The red lines denote flavonoid-mediated inhibition of specific biomolecules.

Nitric oxide (NO), a gaseous free radical produced by activated Mφs, can destroy bacteria, viruses, protozoa, and tumor cells. However, excessive NO production may cause tissue damage and inflammation [144]. In the context of CAC, during phase I, Mφs and neutrophils produce ROS to target cancer cells by inducing lethal DNA mutations, initiating cell death pathways such as caspase-mediated apoptosis, and inhibiting the activity of immunosuppressive cells [145] (Figure 5).

Dietary flavonoids such as quercetin and baicalin reduce oxidative stress by inhibiting iNOS expression and myeloperoxidase (MPO) activity and by scavenging peroxynitrite radicals [146] (Table 3). In UC inflammation, these actions may limit cell damage and restore tissue homeostasis. This antioxidant property is also relevant in early CAC, where ROS trigger pathways such as the NF-κB and STAT3 pathways [147], which influence cell transformation and survival.

In advanced stages of CRC, ROS contribute to the formation of a tumor microenvironment characterized by the presence of Tregs, M2 Mφs, and anti-inflammatory cytokines. Increased levels of ROS in cancer cells can influence the initiation and progression of cancer; however, excessive concentrations of ROS can also be harmful to cells [148]. In this stage, cancer cells develop mechanisms of resistance to oxidative stress that involve multiple pathways to activate the redox-sensitive transcription factors NF-κB, Nrf2, c-jun and HIF-1, resulting in increased expression of molecular antioxidants such as superoxide dismutase (SOD), catalase, thioredoxin and the antioxidant system γ-glutamylcysteinyl glycine (GSH). This redox adaptation can increase tolerance to stress and exogenous insults, decrease apoptotic performance, and improve DNA repair [149]. In addition, alterations in the elimination of ROS enzymes, such as GSH, significantly affect the metabolism of alkylating agents [150].

Quercetin and EGCG, among other flavonoids, can increase the activity of glutathione peroxidase (GPx) and catalase (CAT) and stimulate the production of reduced GSH and SOD in cells, which help reduce the levels of malondialdehyde (MDA) (Table 3) [151]. This increase in antioxidant activity could be counterproductive in the advanced stages of CRC, promoting mechanisms of resistance to oxidative stress and reducing the effectiveness of chemotherapy and radiotherapy treatments. Thus, flavonoid administration at this stage should be approached cautiously [145].

### 4.5. Flavonoids Negatively Regulate Eicosanoids

Eicosanoids derived from arachidonic acid via cyclooxygenase (COX) and lipoxygenase (LOX) enzymes play crucial roles in colon inflammation, influencing the activity of immune cells such as Mφs, neutrophils, T cells and DCs [152].

**Figure 6:**
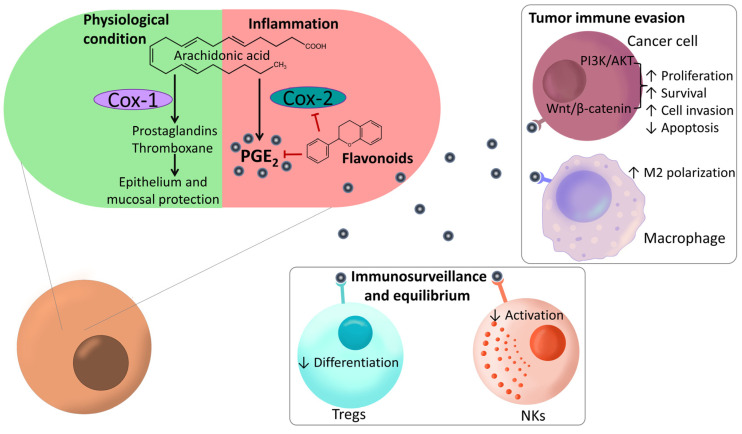
Action of flavonoids on the COX-2/PGE2 axis and the effect of prostaglandin E2 (PGE2) on immunological tumor evasion and immunosurveillance. ↑ indicates upregulation, and ↓ indicates downregulation by flavonoids on the indicated cell or biological process. The black arrows illustrate the endogenous biological pathways and signaling cascades of Cox-1 and Cox-2 in physiological or inflammation conditions respectively. The red lines denote flavonoid-mediated inhibition of specific biomolecules.

Sustained COX-2 production in UC promotes CAC by inhibiting apoptosis, increasing angiogenesis and invasiveness, and converting procarcinogens into carcinogens [153]. Throughout CRC progression, eicosanoids fuel inflammation, ROS generation, and DNA mutation in colonic cells. In established CAC, PGE2 promotes cancer growth, survival, invasion, and therapy resistance through the activation of PI3K/AKT and Wnt/β-catenin signaling, as well as through the inhibition of apoptosis [154]. The COX-2/PGE2/EP2–EP4 axis is also associated with increased Treg-mediated suppression of CD8^+^ and Th1 responses [155] (Figure 6).

Flavonoids like apigenin and quercetin counteract this axis by inhibiting COX-2 and reducing PGE2 levels, making them promising agents for both prevention and adjunctive therapy in CAC, although their dual effects on other pathways must be carefully considered (Table 3).

## 5. Discussion

CAC is the third leading cause of cancer death worldwide and the main cause of death in Mexico [156]. Although the mortality rate from colon cancer is gradually decreasing in Western countries, the incidence and mortality rates are increasing in Latin America [157]. In these regions, CAC could be prevented if accessible therapeutic strategies could be established.

Flavonoids regulate intestinal inflammation; therefore, a comprehensive understanding of their effects is essential for the development of possible therapeutic strategies against IBD and CAC development.

Our analysis underscores that flavonoids effects are complex, and often bidirectional, suggesting that inappropriate use could lead to adverse rather than clinical benefits. Importantly, the heterogeneity of the available literature does not merely reflect experimental variability but rather highlights that flavonoids represent a structurally and functionally diverse group of compounds whose biological effects cannot be interpreted as uniform or interchangeable. While preclinical evidence suggests that flavonoids may mitigate UC and CAC, many of these data stem from in vitro systems or animal models that use specific doses and protocols that do not necessarily mirror human exposure or clinical practice [158]. This gap is particularly evident in large-scale epidemiological studies, which often report inconsistent or even null associations between total dietary flavonoid intake and CRC risk, reporting that the effect depends strictly on the type of polyphenol administered [159,160].

This discrepancy can be attributed to several factors, most notably the relevance of dosage and treatment duration [161]. Many preclinical studies utilize high concentrations that are difficult to achieve through a standard human diet or even oral supplementation, given the low bioavailability and rapid metabolism of these compounds in the gastrointestinal tract [162]. Moreover, the administration route, often through intraperitoneal injection or gavage in murine models, does not always reflect a realistic clinical approach for human prevention, where oral intake and interaction with the colonic microbiota are determinants. Consequently, while in vivo evidence is robust, there remains a notable lack of large-scale clinical trials confirming these effects in humans, with most clinical data limited to small-scale safety pilots rather than long-term efficacy against CAC progression.

A critical comparison of the literature further revealed that not all flavonoids exert comparable immunological effects across disease stages. For example, compounds such as quercetin and luteolin consistently demonstrate anti-inflammatory activity during the early phases of colitis by limiting NF-κB activation and proinflammatory cytokine production [163]. In contrast, other flavonoids, such as genistein or even the same compounds administered at higher doses or during later stages, have been reported to attenuate antitumor immune mechanisms. By hindering IFN-γ–dependent responses or STAT1 signaling, these interventions may inadvertently favor immune evasion once a malignancy is established. Furthermore, recent evidence suggests that flavonoids can act as potent adjuvants in tumor immunotherapy by reversing the immunosuppressive microenvironment in several types of cancer, including CRC [164].

The divergent outcomes reported across studies are not necessarily contradictory; instead, they reflect the intricate interplay between flavonoid structure, molecular targets, dosing regimens, and, crucially, the timing of intervention. These findings emphasize that future research must account for the specific stage of CAC development to scientifically justify the use of flavonoids. By providing a rigorous bibliographic foundation, this review supports the move toward a more rational and chronological clinical application of flavonoids in treating colon-associated inflammatory processes.

## 6. Conclusions and Future Directions

The dual role of flavonoids does not reflect contradictory biological actions but rather distinct immunological requirements across disease stages. During active colitis and early inflammation-driven tumorigenesis, flavonoids may reduce mucosal damage and chronic inflammation, thereby lowering the risk of CAC development. However, once malignant transformation has occurred, particularly in established or advanced tumors (stages II and III), excessive attenuation of inflammatory signaling may impair immune surveillance, facilitate immune escape, and potentially interfere with the efficacy of anticancer therapies.

On the basis of the evidence discussed, flavonoids may be considered complementary agents for the management of inflammatory colitis and UC to reduce the risk of CAC. Conversely, their use in patients with established CAC should be approached with caution and cannot be recommended without stage-specific evaluation. These findings highlight the need for future studies that are explicitly designed to address disease stage, immune context, and treatment timing to define rational and safe therapeutic strategies involving flavonoids.

## Figures and Tables

**Table 1:** Flavonoid classification and chemical structure [4].

Compound Class	Key Structural Features	Chemical Structure
Anthocyanins	Glycosides derived from polyhydroxy and polymethoxy substituents within the flavylium core	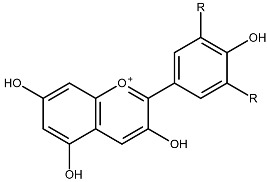
Flavonols	Specific substitutions on rings A and B, hydroxyl groups at positions 5 and 7 on ring A linked to a three-carbon chain	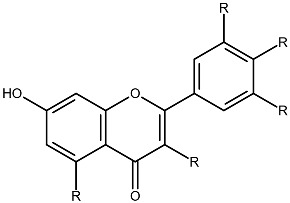
Flavones	4H-chromen-4-one backbone with a phenyl group at position 2; commonly 7-O-glucosides	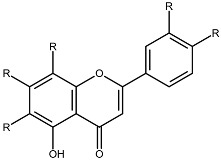
Flavanones	Fully saturated C ring, lacking a double bond between C2 and C3 positions	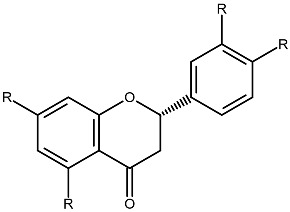
Isoflavones	3-phenylchromen-4-one structure, structural isomers of flavones, phenyl group at position 4	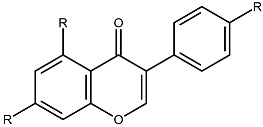
Flavan-3-ols	Hydroxyl group at position 3 on the C ring, absence of a double bond between positions 2 and 3	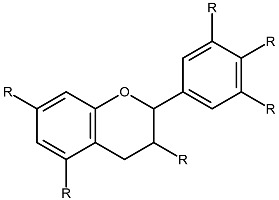

## Data Availability

No datasets were generated or analyzed during the current study.

## Abbreviations

AKT	Protein Kinase B
Ahr	Aryl hydrocarbon receptor
BCl2	B-cell lymphoma 2
CRC	colorectal cancer
CAC	colitis-associated cancer
CAT	Catalase
CDK	cyclin-dependent kinase
CLR	C-type lectin receptor
COX	cyclooxygenase
CXCL	chemokine ligand with CXC motif
DCs	dendritic cells
EGCG	epigallocatechin gallate
EP	E-prostanoid receptor
ERK	extracellular signal-regulated kinase
GPx	glutathione peroxidase
GSH	γ-glutamylcysteinyl glycine
HClO_2_	chlorous acid
HIF-1	hypoxia-inducible factor 1
IBD	inflammatory bowel disease
IL	interleukin
INF	interferon
iNOS	inducible nitric oxide synthase
JAK	janus kinase
JNK	Jun N-terminal kinase
KC	keratinocytes
LOX	lipoxygenase
MAPK	mitogen-activated protein kinase
Mφs	macrophages
MDA	malondialdehyde
MDSCs	myeloid-derived suppressor cells
MIP	inflammatory protein of Mφ
MPO	myeloperoxidase
NF-κB	nuclear factor kappa B
NKs	natural killer cells
Nrf2	nuclear factor erythroid 2-related factor 2
NO	nitric oxide
PGE2	prostaglandin E2
PI3K	phosphoinositide 3-kinase
PD-L1	programmed cell death 1 ligand 1
ROS	reactive oxygen species
SOD	superoxide dismutase
STAT	signal transducer activation and activator of transcription
TAMs	Mφ associated with tumors
TGF	transforming growth factor
Th1	T helper 1 cells
Th2	T helper 2 cells
Th17	T helper 17 cells
TLR	Toll-like receptor
TNF	tumor necrosis factor
Tregs	regulatory T cells
UC	ulcerative colitis
ZO-1	Zonula occludens-1
